# Protein self-assembly onto nanodots leads to formation of conductive bio-based hybrids

**DOI:** 10.1038/srep38252

**Published:** 2016-12-06

**Authors:** Xiao Hu, Chenbo Dong, Rigu Su, Quan Xu, Cerasela Zoica Dinu

**Affiliations:** 1 Department of Chemical and Biomedical Engineering, West Virginia University, WV, USA; 2 State Key Laboratory of Heavy Oil Processing, China University of Petroleum (Beijing), Beijing, China

## Abstract

The next generation of nanowires that could advance the integration of functional nanosystems into synthetic applications from photocatalysis to optical devices need to demonstrate increased ability to promote electron transfer at their interfaces while ensuring optimum quantum confinement. Herein we used the biological recognition and the self-assembly properties of tubulin, a protein involved in building the filaments of cellular microtubules, to create stable, free standing and conductive sulfur-doped carbon nanodots-based conductive bio-hybrids. The physical and chemical properties (e.g., composition, morphology, diameter etc.) of such user-synthesized hybrids were investigated using atomic and spectroscopic techniques, while the electron transfer rate was estimated using peak currents formed during voltammetry scanning. Our results demonstrate the ability to create individually hybrid nanowires capable to reduce energy losses; such hybrids could possibly be used in the future for the advancement and implementation into nanometer-scale functional devices.

Nanowires have numerous interesting properties such as direct band gaps resulted from their quantum confinement^1^, or precise control of their composition^2^. Geometrical anisotrophy, increased surface to volume ratios, and dipolar magnetic properties are function of the nanowires shape^3^ and were shown to influence their ability to transfer energy at interfaces, mostly because of the confinement of the electrons or the energy levels these electrons occupy^4^. For instance, Fuhrmann *et al*.^5^ reported a lithography method combined with molecular beam epitaxy, while Choi *et al*.^6^ used an interface lithography technology combined with catalytic etching to create silicon nanowires arrays with demonstrated interfacial electron transfer. In addition, chemical vapor deposition (CVD) was employed to fabricate germanium-based nanowires at 275 °C^7^. However, such methods only allow for a limited control of the nanowire’s structure–property relationships thus limiting its implementation in nano-functional devices. Further, the growth in porous templates typically produces polycrystalline materials of large diameters thus not offering the small size necessary to generate the quantum confinement effect^8^. Moreover, the precursors used in the CVD process for instance, may be highly toxic (nickel tetracarbonyl), explosive (diborane), or corrosive (silicon tetrachloride)^9^, thus posing a logistical burden to both the environment and the user. Lastly, the tedious and time consuming steps that the lithography technology requires restrict the large scale synthesis of such nanowires^10^.

Combining atoms or individual nanostructures might hold the promise for the next generation of alternative strategies aimed to create bottom-up nanowires to be integrated in synthetic nanodevices. As such, carbon nanodots (C-dot), i.e., photo-stable nanomaterials made out of carbon^11^, have generated considerable interest for nanowires formation due to their ability to photo-induce electron transfer at their interfaces^12^. Claims about effective charge-transfer properties also rendered C-dots usage as viable candidates for nanowire-based hybrid formation that could possibly reduce energy losses while ensuring efficient transport of electrons^13^. However, so far reports only showed C-dots applications in photocatalysis^14^,^15^, storage and transport of electrons^16^,^17^, lighting systems^18^,^19^, bioimaging^20^,^21^, nanosensors^22^,^23^ with improved surface-enhanced Raman scattering^24^,^25^ or single-particle spectroscopy with photoluminescence ability varying with C-dot sizes^15^. For instance Kang *et al*.^26^ have demonstrated that iron oxide (Fe_2_O_3_)-C-dot-based composites have a higher photocatalytic activity as well as continuous and broad absorption in the 550–800 nm range relative to bare Fe_2_O_3_ nanoparticles. Bao *et al*.^27^ found that the red shift in C-dot emission capability was independent of its size. Properties such as strong photoluminescence resulted from the recombination of photo-generated charges on the defect centers of the C-dot^28^ were exploited for the creation of the next generation of fluorescent probes capable to detect mercury in solution^29^,^30^ or for Fe^3+^ detection^31^. Complementarily, the high aqueous solubility, nanoscale size which resemble biological agents such as viruses, bacteria or pathogens^32^, as well as their increased biocompatibility^33^ were explored for drug delivery applications^34^,^35^ or for ultra sensitive detection of tumor markers such as prostate protein antigen^36^. However, before the C-dots integration in functional devices is to be achieved, charge-transfer properties at their interfaces need to be correlated to their ability to form conductive and controllable geometries.

Several strategies attempted to create nanometer-scale integrated circuits to be used for optical devices^37^ by considering closely spaced metallic nanodots as a possible solution to reduce energy losses at interfaces^38^. For instance, Nomura *et al*.^39^ fabricated a nanodot coupler from closely spaced structures and demonstrated its ability to convert propagating far-field to an optical near-field light^40^. The high efficiency recorded in such an approach was a result of the coupling of the scattering at the metallic nanodot interfaces^41^. Furthermore, when nanodot arrays were used as substrates, localized surface plasmon resonance detection of antigen-antibody binding was also demonstrated, with the analyses showing that the extinction peaks intensities associated with the specific detection were dependent on the height of the nanodot arrays as well as influenced the array’s ability to detect the light transmitted at its interface^42^. However, while these examples demonstrate the ability to create individual surface nanodot-based structures, they do not provide the proof-of-concept that the scaffold-addressable nanodots forming such structures are capable to reduce energy losses or advance nanometer-scale implementation of nanowires in functional devices.

We aim to create the next generation of hybrid nanowires through self-recognition and self-assembly properties of biological molecules responsible for cell structure formation^43^. Specifically, using user-synthesized sulfur-doped C-dots templates and tubulin, the precursor of microtubules cytoskeletal filaments, we demonstrated the ability to control the synthesis and stability of bio-based hybrid nanowires as well as their conductivity, to thus help evaluate their forecast for implementation in the next generation of functional nanodevices with nanometer size distribution and high quantum yield.

## Materials and Methods

### Synthesis and characterization of the sulfur-doped carbon nanodots

Sulfur-doped carbon nanodots (S-doped C-dots) were synthesized using a hydrothermal method^31^. Briefly, 0.1 M 25 mL sodium citrate and sodium thiosulfate solutions (Tianjin Guangfu Technology Development Co., Ltd., China) were added into a 50 mL Teflon-lined stainless steel autoclave (Fuzhou Keleisi Test Equipment Co., Ltd., China) and the autoclave was incubated at 200 °C for 6 h. The resulting products were filtered through a 0.22 μm filtration membrane (Spectrum Laboratories, Inc. USA).

Such user-synthesized S-doped C-dots were further characterized using both morphological and structural-based techniques. Specifically, for sample size and morphology analyses, contact mode Atomic Force Microscopy (AFM, Asylum Research, USA) with a silicon nitride tip (TR-400PB, Asylum Research, USA) in ambient or in solution was used. The trigger force was kept constant at 3 nN while the spring constant of the cantilever was measured before each experiment using established methods^44^. For these analyses, mica sheets were first washed with deionized water (DI water), ethanol (90%, Fisher Scientific, USA), and again with DI water, and subsequently dried under vacuum overnight and at the room temperature. The cleaned mica was subsequently functionalized with 3-aminopropyltriethoxysilane (APTES, Fisher Scientific, USA; 20 μL, 99%) through incubation at room temperature for 15 min; any remaining solution was removed under vacuum. Solution of C-dots in DI water (20 μL; 0.05 mg/mL) was dropped onto the APTES functionalized mica and either dried under vacuum at room temperature for 3 h or used directly for imaging in contact mode.

Elemental composition of the user-synthesized S-doped C-dots was evaluated using Energy Dispersive X-ray (EDX, JEOL JSM-7600F Scanning Electron Microscope, USA) and X-ray Photoelectron (XPS, Physical Electronics PHI 5000 VersaProbe XPS/UPS, USA) spectroscopies. The EDX was operated at 20 kV while the pass energy for XPS was maintained at 117.4 eV with a 0.5 eV step for survey scan or 23.5 eV and 0.05 eV for detailed scan respectively.

Attenuated Total Reflectance Fourier Transform Infra-Red Spectrometry (ATR-FTIR; Digilab FTS 7000/UMA 600, USA) allowed for specific functional groups associated with the user-synthesized S-doped C-dots to be identified. The FTIR scans where performed in the 700 to 4000 cm^−1^ range using a scan speed of 20 kHz and a 10 reflection diamond ATR. The FTIR software packages incorporated an ART correction algorithm for the resulted spectra.

### Synthesis of microtubule hybrid bio-nanowires or S-doped C-dots hybridized microtubule

Microtubules were synthesized according to established protocol^45^ from free tubulin incubated in a microtubule polymerization solution. Briefly, the microtubule polymerization solution was obtained by vortexing 5 μL 100 mM magnesium chloride (MgCl_2_, Fisher Scientific, USA), with 6 μL dimethyl sulfoxide (DMSO, 99.7%, Fisher Scientific, USA), 5 μL 25 mM guanosine-5′-triphosphate (GTP, Sigma, USA) and 9 μL BRB80 buffer (formed from a mixture of 80 mM piperazine-N,N’-bis(2-ethanesulfonic acid buffer, 1 mM MgCl_2_ and 1 mM ethylene glycol tetraacetic acid (EGTA), pH 6.8; all reagents were purchased from Fisher Scientific, USA).

To initiate microtubule polymerization, 2.5 μL polymerization solution was injected into 10 μL of 4 mg/mL biotin-tubulin (Cytoskeleton Inc, USA) and the mixture was incubated at 37 °C for 30 min. To stabilize the resulting microtubules, the solution was dispersed in 1 mL BRB80 buffer containing 10 μM taxol (Fisher Scientific, USA). The stabilized microtubules were kept at room temperature for experimental usage.

For the bio-hybrid synthesis also called hybrid microtubule, first biotin-tubulin-S-doped C-dots-conjugates were formed using non-specific binding of biotin-tubulin onto S-doped C-dots scaffolds^46^. Briefly, 1 μL 6 mg/mL synthesized S-doped C-dots were injected into a 600 μL eppendorf tube containing 5 μL of 4 mg/mL biotin-tubulin and the mixture was incubated for 2 h at 200 rpm in an ice bath. Hybrids were assembled from the conjugates and free biotin-tubulin in solution as previously described. Briefly, 5 μL of 4 mg/mL free biotin-tubulin was mixed with the biotin functionalized tubulin- S-doped C-dots conjugates and an additional 2.5 μL microtubule polymerization solution, and subjected to 37 °C for 30 min. When time elapsed, the hybrids were stabilized in BRB80 buffer containing 10 μM taxol and used for the experiments.

### Biological-based sample AFM analyses

The biological-based samples (either microtubules, S-doped C-dots hybridized microtubules or biotin-tubulin) were analyzed for their morphology and physical characteristics using the AFM machine previously introduced (see quantum dots section). For this, 20 μL of the sample with or without 0.5% glutaraldehyde (Fisher Scientific, USA) was dropped onto the APTMS functionalized mica surface (see protocol above) and incubated for 1 h at room temperature. After the incubation, the surface was washed with 40 μL BRB80 buffer containing 10 μM taxol for at least 3 times.

The AFM images were collected immediately after the washing step using contact mode AFM in solution as previously described (see quantum dots section). For these measurements the cantilever’s spring constant was calibrated using the thermal noise method^44^; the scan rate of the tip was fixed at 0.5 Hz for all the experiments. At least 50 (20 for microtubule, 24 for S-doped C-dots hybridized microtubules or 6 biotin-tubulin) biological-based samples have been investigated to collect their morphology and for diameter analyses. The height of the samples (i.e., S-dots, microtubules, or hybrids) was estimated by running linear cross-sections at different points.

For the microtubule diameter, we analyzed at least 72 individual microtubules collected from 20 independent images. The diameter distribution of S-doped C-dots hybridized microtubules was collected from 24 independent AFM images that included 93 individual microtubules. Lastly, no deconvolution of the tip was considered necessary since previous analyses have showed that the width of the small objects as collected using AFM is often distorted due to the combination of the tip and sample geometries, while the height is not^47^,^48^.

### Fabrication of the modified gold electrodes

To prepare for voltammetry measurements, 4 gold electrodes (CH Instrument Inc., USA) were first cleaned by immersion in Piranha solution (containing 96.4% sulfuric acid, H_2_SO_4_, Fisher Scientific, USA) and 30% hydrogen peroxide (H_2_O_2_, Fisher Scientific, USA) in a 3:1 (v:v) for 10 min, then rinsed thoroughly with the DI water^49^. Subsequently, the gold electrodes were polished successively with 1.0, 0.3 and 0.05 μm α-alumina (CH Instrument Inc., USA) powders and again rinsed with DI water to remove any impurities resulted from such polishing.

All the clean gold electrodes were further activated by applying electrochemical oxidation-reduction cycles in H_2_SO_4_^50^; specifically, the potential was cycled from −200 to 1600 mV (vs. Ag/AgCl) at a scan rate of 50 mV/s in 0.5 M H_2_SO_4_ on a VersaSTAT 3 potentiostat/galvonostat (Princeton Applied Research, USA). The process was repeated until the cyclic voltammetry graph read for each of the electrodes became stable. Upon the treatment, the electrodes were rinsed thoroughly with the DI water and then used for sample functionalization analyses. Briefly, for the chitosan functionalization, 10 μL 1% (wt) chitosan in BRB80 containing 10 μM taxol was incubated onto the surface of one of one electrode, with the incubation being performed under vacuum and overnight. For the S-doped C-dots, microtubules or S-doped C-dots hybridized microtubules functionalization of the remaining 3 electrodes respectively, 10 μL 1% (wt) of chitosan in BRB80 containing 10 μM taxol was mixed with either 10 μL S-doped C-dots (0.5455 mg/mL), microtubules (32 nM) or S-doped C-dots hybridized microtubules (32 nM), and subsequently the solution was dropped onto each of the activated/clean electrodes and incubated overnight, all under vacuum.

### Electrochemical measurement

Differential pulse voltammetry (DPV) and electrochemical impedance spectroscopy (EIS) were performed on the VersaSTAT 3 potentiostat/galvonostat. For the DPV, the voltage ranged from 0.2 V to −0.6 V with a 50 ms pulse period, 49 ms pulse width, 50 mV pulse height and 1 mV pulse potential increment respectively. The DPV analyses were performed using 1.5 × 10^-4^ M methylene blue (MB, Fisher Scientific, USA) in BRB80 buffer containing10 μM taxol and 10 mM sodium chloride (NaCl, Fisher Scientific, USA). For the EIS, the frequency ranged from 0.01 Hz to 100000 Hz, while the amplitude of 5 mV was maintained constant. The EIS analyses have been performed in 50 mM potassium ferricyanide (K_3_Fe(CN)_6_; Fisher Scientific, USA) in BRB80 buffer containing 10 μM taxol. The choice of feerricyanide was based on previous literature reports which showed that this agent is suitable for evaluating electron transfer at an electrode surface^51^,^52^. The supporting electrolyte used in our experiments is 10 mM NaCl. At least 6 experiments have been performed for each of the samples being reported.

## Results and Discussion

Herein we hypothesized that hybrid nanowires containing carbon nanodots (C-dots) can be obtained by using self-assembly and self-recognition-based biological processes. Further, we hypothesized that such hybrids’ characteristics (both physical and chemical) can be manipulated to increase hybrid’ operational stability and thus extend its operation for possible nanometer-based device implementation.

To demonstrate our first hypothesis, we synthesized nanowire-based templates of sulfur-doped carbon nanodots (S-doped C-dots) using a hydrothermal method and a mixture of sodium citrate and sodium thiosulfate solutions. Previous analyses showed that such S-doped C-dots have a high fluorescence quantum yield (67%) and could be used as fluorescent probes for iron detection^31^. User-synthesized S-doped C-dots were evaluated for their physicochemical properties using atomic force microscopy (AFM), Fourier Transform Infrared Spectroscopy (FTIR), Energy-dispersive X-ray (EDX) and X-ray photoelectron (XPS) spectroscopy.

**Figure 1:**
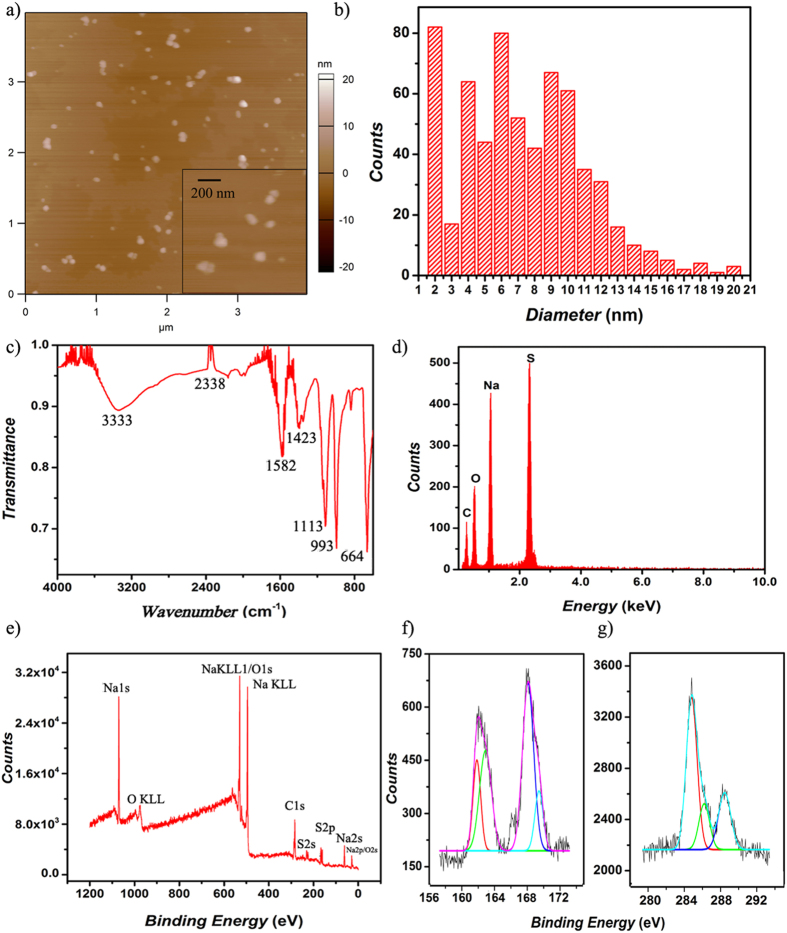
Surface characterization of the S-doped C-dots. (**a**) Representative Atomic Force Microscopy (AFM) analyses that allow for individual or conglomerates of nanodots visualization. AFM insert in the scale of 1 μm × 1 μm allows for particle size analyses. (**b**) The diameter distribution of the S-doped C-dots as resulted from AFM evaluations. (**c**) Fourier Transform Infrared Spectroscopy (FTIR) of the S-doped C-dots allows for identification of surface functional groups, while Energy Dispersive X-Ray (EDX) analysis (**d**) reveals the elemental composition of the S-doped C-dots. (**e**) The X-ray Photoelectron Spectroscopy (XPS) survey graph of the S-doped C-dots. (**f**) High-resolution S_2p_ XPS spectra of the S-doped C-dots. (**g**) High-resolution C_1s_ XPS spectra of the S-doped C-dots.

AFM analyses performed in visual fields of 4 μm × 4 μm allowed for both S-doped C-dots as well as any resulting conglomerates identification. Well-separated and quasi-spherical nanodots are shown in Fig. 1a insert, with the diameter distribution being shown in Fig. 1b. Analyses revealed S-doped C-dots of a rather narrow size (between 2 to 10 nm) and an average of about 7.3 nm (about 620 nanodots were analyzed). More than 81% of the nanodots showed diameters of less than 10 nm indicating controlled synthesis that allowed for tight size selectivity. AFM analyses of S-doped C-dots sizes were also confirmed using high resolution transmission electron microscopy (HRTEM- Supplementary Information, Figure S1).

FTIR helped evaluate the chemistry of the user-synthesized S-doped C-dots to further ensure the feasibility of the method used for lab synthesis. Figure 1c identifies a broad peak at 3333 cm^−1^ which was attributed to the stretching vibration of the O–H bonds in the carboxylic acid (-COOH) group and/or intercalated water molecules obtained during the S-doped C-dots synthesis^53^. The peak at around 2338 cm^−1^ was associated with the carbon dioxide (CO_2_) vibration^54^ which could presumably be due to the mismatch between the CO_2_ concentration in the atmosphere during the baseline acquisition and the atmospheric CO_2_ concentration during the measurements, while the peak at 1582 cm^−1^ represents the fingerprint region of the carbonyl group (C=O), carbon oxygen bond (C-O) and hydroxyl (-OH) groups^55^. The additional peak observed at 1423 cm^−1^ was ascribed to the –COOH group^56^ while the peak at 1113 cm^−1^ was attributed to the C–O stretching and the association of the -OH bonds^57^. Lastly, the peak at 993 cm^−1^ was attributed to C–O stretching and deformation^57^, C–O–C bonds or free sulfite (SO^3−^)^58^ respectively.

EDX (Fig. 1d) and XPS (Fig. 1e) analyses helped evaluate the elemental composition of the user-synthesized S-doped C-dots and confirmed the presence of the starting elements used in such synthesis, namely C, O, Na and S. A detailed component analysis of their % distribution is shown in Table 1. High resolution XPS (Fig. 1f,g) complemented the FTIR spectrum and confirmed the presence of sulfur (S_2p_), with two peaks, one at around 162.11 eV and the second one at 163.29 eThe peaks were attributed to the sulfion (S^2−^)^59^ and sulfite ion (SO_3_^2−^)^60^ respectively, as resulted from the synthesis process. Complementarily, the C1s spectra is shown in Fig. 1g and reveals characteristics associated with the C-C (284.7 eV), C-H (284.7 eV)^61^, C-O (285.87 eV)^62^ and C=O (288.43 eV)^63^ respectively.

**Figure 2:**
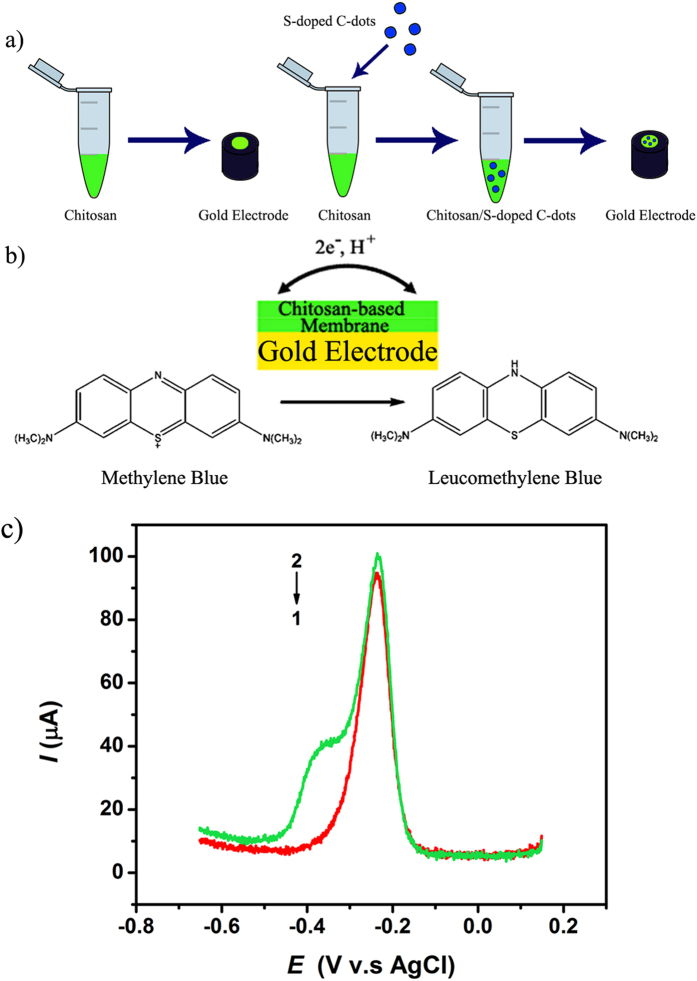
(**a**) Representative schematic of the chitosan-based membrane formation at the gold electrode interface. (**b**) The reduction reaction of the methylene blue (MB) onto the electrode surface. (**c**) Differential Pulse Voltammetry (DPV) graph of the MB on 1) chitosan/gold electrode (red), 2) S-doped C-dots/chitosan/gold electrode (green).

Secondly, we evaluated the electrochemical properties of the user-synthesized and above characterized S-doped C-dots using differential pulse voltammetry (DPV; Fig. 2). For this, chitosan, a linear polysaccharide was used to suspend the S-doped C-dots to form a membrane onto the DPV electrodes; the electrochemical reaction efficiency was based on the rate of ion generation upon interaction with methylene blue (MB) (Fig. 2a). The organic dye MB was chosen based on previous analysis that showed the dye to be an efficient electrochemical agent capable to allow for changes in intensity as resulted from the reduction and oxidation induced currents to be quantified^64^. Previous studies have also showed that charge-transfer properties are observed when using electron-donating C-dots and electron-accepting perylenediimides^65^; studies demonstrated that C-dots can be integrated in nafion nanocomposite to enhance the detection sensitivity of an electrochemical immunosensor^66^. Further, analyses revealed that chitosan is a suitable carrier material for electrochemical biosensors^67^, with Sun *et al*.^68^ using chitosan containing embedded acetylcholinesterase to probe kinetics at interfaces. Complementarily, Li *et al*.^69^ developed a chitosan-embedded nonenzymatic glucose sensor that was based on glassy carbon electrode (GCE) coated with platinum hollow nanoparticle chains dispersed onto porous gold nanoparticles. Lastly, Xu *et al*.^70^ entrapped hemoglobin into a graphene and chitosan composite film for direct electrochemical behavior on a GCE.

Compared with the chitosan only, the user-synthesized S-doped C-dots embedded in the chitosan membrane provided a conductive microenvironment that facilitated electron transfer as shown by the increase of the peak current of this membrane relative to the only chitosan-based membrane (Fig. 2b). Specifically, peaks centered at about −0.22 V were observed for both membranes (with and without the embedded S-doped C-dots respectively; Fig. 2c). Additionally, a new peak was observed for the S-doped C-dots embedded in the chitosan membrane, with this peak being centered at about −0.36 V (about −0.58 V vs. standard hydrogen electrode) and attributed to the redox potential of the sulfite (SO_3_^2−^)^71^. The relative low intensity of this peak when compared with the one centered at −0.22 V was presumably due to the low concentration of the SO_3_^2−^ resulted from the S-doped C-dot synthesis as confirmed by the spectroscopy analyses included above.

The electron transfer rate at the S-doped C-dots embedded in the chitosan membrane was estimated using the Laviron’s equation^72^,^73^ (**Eq: 1**) and compared with the transfer rate at the chitosan membrane only. Specifically,


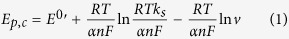


with the cathodic peak potential (E_p,c_) being function of the formal potential (E_0_), α the cathodic electron transfer coefficient, the scan rate(v) with the symbols R, T and F representing the ideal gas constant, absolute temperature and Faraday constant respectively, and n representing the number of electrons involved in the redox reaction.

Analyses showed that the electron transfer coefficient was increased from 3.309 to 4.618 for the S-doped C-dots-containing membrane when compared to the chitosane only membrane, thus indicating a larger electron transfer at the C-dots-containing membrane interface.

Thirdly, we demonstrated the ability to fabricate hybrid bio-nanowires containing S-doped C-dots by using self-assembly and self-recognition of polymeric biomolecules, i.e. tubulin into a two-step strategy. Tubulin is a globular protein that assembly to form a microtubule cytoskeletal structure in the cell, which serves as filament for cell-based transport or for template of cell division^74^. Previous reports have showed that tubulin could be templated onto carbon nanotubes to form hybrid systems with tubular shape^75^ that could alter cell mechanics^76^ and induce toxicity^77^.

In our first step, we formed tubulin-S-doped C-dots conjugates by physical adsorption of free tubulin onto the user-characterized S-doped C-dots^46^. Our theoretical analyses based on the amount of protein used for loading (see materials and methods) and the size characteristics of the user-synthesized S-doped C-dots showed that the protein was adsorbed onto the nanodots to a loading of about maximum 4 tubulins per individual S-doped C-dot. In the second step, the templated tubulin was combined with additional free tubulin to form hybrid bio-nanowires or S-doped C-dots-based nanowires. This was achieved through simply exploiting tubulin known ability to polymerize into the microtubular structure in the presence of guanosine triphosphate^45^.

**Figure 3:**
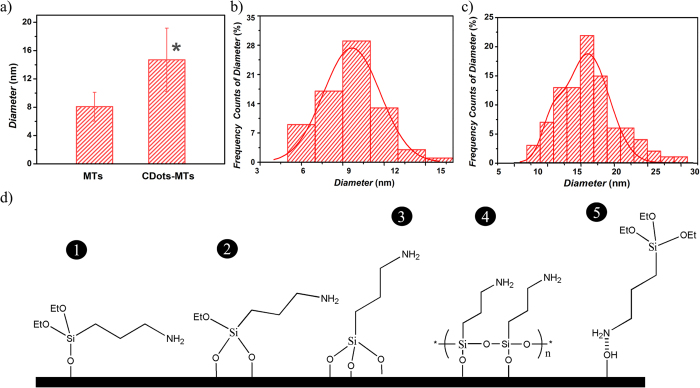
(**a**) Average diameter of microtubules and the S-doped C-dots hybridized microtubules as measured using cross-sectional analyses. (**b**) The diameter distribution of 72 individual microtubules collected from 20 independent images. (**c**) The diameter distribution (as measured using cross-sectional analyses) of S-doped C-dots hybridized microtubules and collected from 24 independent AFM images that included 93 individual microtubules. (**d**) Different orientations or combination of such orientations of the APTES molecules on the mica surface lead to different geometries and size distribution of the microtubules.

Synthesized S-doped C-dots-based microtubule hybrids were evaluated for their morphology, integrity and stability using (3-aminopropyl)triethoxysilane (APTES) functionalized mica surfaces and AFM analyses in solution, and compared to control microtubules polymerized only from free tubulin. Figure 3a depicts the average diameter of the hybrid microtubules when compared to microtubules used as controls respectively. Analyses showed that microtubules had an average diameter of about 8 nm, while the diameter of the S-doped C-dots hybrid microtubules was significantly higher, i.e., 14 nm (student’s T-test p* < 0.05). About 30% (i.e., 72 microtubules) were about 10 nm while no more than 5% of them had a diameter larger than 13 nm (Fig. 3b). Figure 3c shows the diameter distribution of the S-doped C-dots hybrid microtubules, ranging from 6 to 31 nm with about 23% (out of the 93 S-doped C-dots hybrid microtubules being analyzed) having an average diameter of about 16 nm.

The observed uneven diameter distributions are presumably due to the non-uniform absorption of the APTES onto the mica surface as well as from the uneven incorporation of the S-doped C-dots onto the hybrid microtubule structure during the template-tubulin polymerization. The first statement is supported by previous analyses of microtubules in solution^78^ which recorded irregular diameter distributions as result of their irregular absorption onto a similarly functionalized mica. Specifically, with APTES possibly assuming 5 orientations^79^ (Fig. 3d) including one (1), two (2) or three (3) adsorbed ethoxy groups, as well as due to the polymerization with multiple ethoxy groups binding to the silanol-terminated silicon (4), or hydrogen bond formation between the NH_2_ group on the APTES molecule and the mica surface (5), APTES would possibly undergo different conformations thus leading to anisotropic geometries onto the mica surfaces. Such geometries could further lead to different polymer’s lengths being exposed above the mica resulting in diverse microtubule embedment and non-uniform distribution of its diameter. Such distributions could possibly also be responsible for the variations recorded for the hybridized bio-nanowires, namely the ones containing the S-doped C-dots-based microtubules.

**Figure 4:**
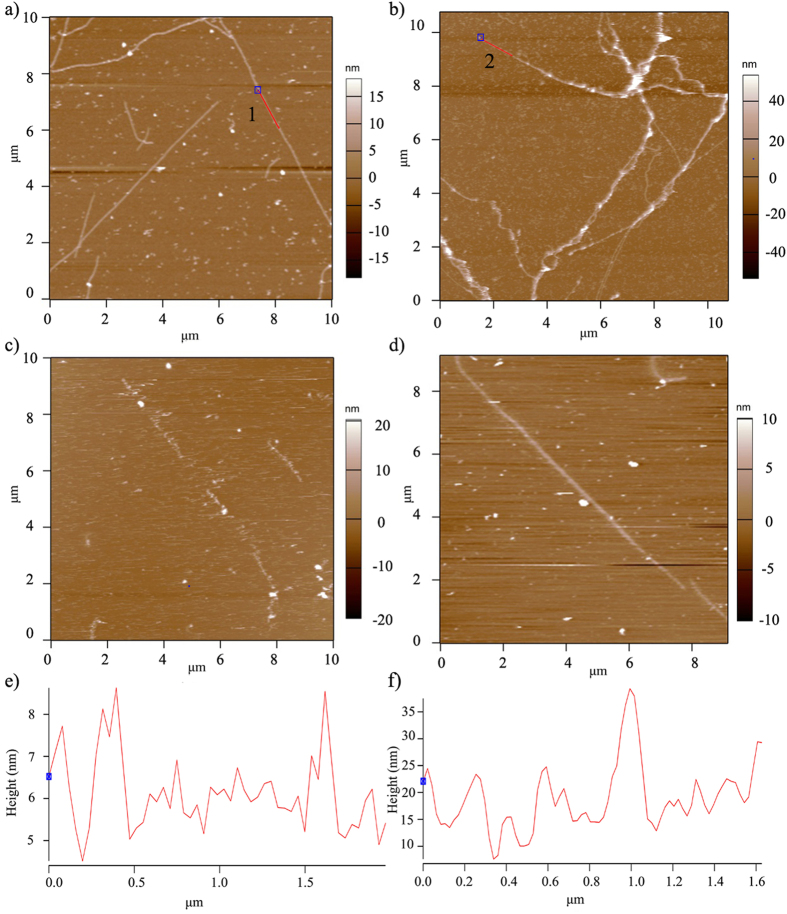
(**a**) Representative morphology AFM analyses of microtubules after crosslinking with glutaraldehyde. (**b**) Representative morphology AFM analyses of S-doped C-dots hybridized microtubules after crosslinking with glutaradehyde. Reduced stability was observed for the microtubules not crosslinking with glutaraldehyde (**c**) while the morphologies of the hybrid microtubules was not affected by the lack of the crosslinking agent (**d**). (**e**) Height distribution of the microtubule (along the red line 1 in Fig. 4a; cross-sectional analyses) or S-doped C-dots hybridized microtubules (along the red line 2 in Fig. 4b; cross-sectional analyses) with the blue square representing the start point (0.0 μm). The line followed the sample morphology.

To demonstrate the second hypothesis, i.e., that the characteristics of such user-formed hybrid bio-nanowires can be manipulated to increase their stability, we first performed morphology analyses of both the microtubule (control) and S-doped C-dots hybrid microtubule crosslinked with glutaraldehyde by using AFM contact mode in solution (Fig. 4). Glutaraldehyde was chosen to stabilize the microtubule and hybrid structures^80^ since previous studies showed that the compound can be used for both inter and inner molecular condensation of biological molecules^81^, with Walt *et al*.^82^ showing that glutaraldehyde forms a Schiff’s base with the lysine residues in a protein, both under acidic or neutral conditions, and Tashima *et al*.^83^ respectively showing that an addition reaction occurs in alkaline condition. Further, glutaraldehyde was previously shown to be used as a suitable agent for microtubule crosslinking known to stabilize its gross structure against air drying or a distilled water challenge^84^ with the cross-linked microtubules showing minimal changes from native properties.

Our analyses showed linear-like geometries with non-uniform transversal height distribution for both the microtubule (Fig. 4a) and its hybrid bio-nanowire counterpart (Fig. 4b) respectively. The morphology of the microtubule was smooth and linear when compared to the entangled and bead-like morphology observed for the hybrid microtubule (Supplementary Information Figure S2). Debris associated with either free tubulin, S-doped C-dots or tubulin-S-doped C-dots conjugates was also observed.

Control experiments performed on microtubules only (i.e., not stabilized with glutaradehyde) showed sample denaturation (Fig. 4c) while no denaturation was observed for the counterpart hybrids bio-nanowire (Fig. 4d). Specifically, the hybrids did not only kept their morphology under AFM scanning conditions, but further, analyses allowed for their optical examination for up to 9 days which was in contrast with the 5 days recorded for their counterparts. This is presumably due to the amino groups in the tubulin protein reacting with the aldehyde groups in the glutaraldehyde to form chemical bonds^85^ that limited microtubule denaturation and thus led to its increased stability. Further, the increased stability of the hybrids can also be due to the unsaturated bonds (C=O and C=C groups) in the S-doped C-dots reacting with the aldehyde groups in glutaraldehyde to limit hybrid denaturation.

Figure 4e,f show the cross-sectional height distributions (red lines e and f in Fig. 4a,b respectively) along the glutaraldehyde stabilized microtubule or S-doped C-dots hybrid microtubule, respectively. The average height based on cross-sectional analysis of the normal microtubule was about 7 nm while the height of the S-doped C-dots hybridized microtubule was about 15 nm with the height distribution of the microtubule seeming more uniform than that of the S-doped C-dots hybrid microtubule.

We also evaluated the electrochemical properties of the S-doped C-dots hybrid microtubule or hybrid bio-nanowires. For this, we first incubated chitosan with the formed hybrids and subsequently used DPV and impedance analyses to assess their conductivity. The consideration for incubating chitosan with the microtubules or hybrids directly is based on previous analyses that showed that interaction of chitosan with proteins forms complexes, mainly through hydrophobic and electrostatic contacts (i.e., function of the protein structural elements^86^) with such interaction inducing protein destabilization. If destabilization would be induced on the tubulin, then no self-assembly or microtubules^84^ formation would be observed; to support this, control experiments in which chitosan was added to only free tubulin were performed (Supplementary Information, Figure S3). The electrodes functionalization with chitosan solution was performed in a manner similar to the one described in Fig. 2a.

**Figure 5:**
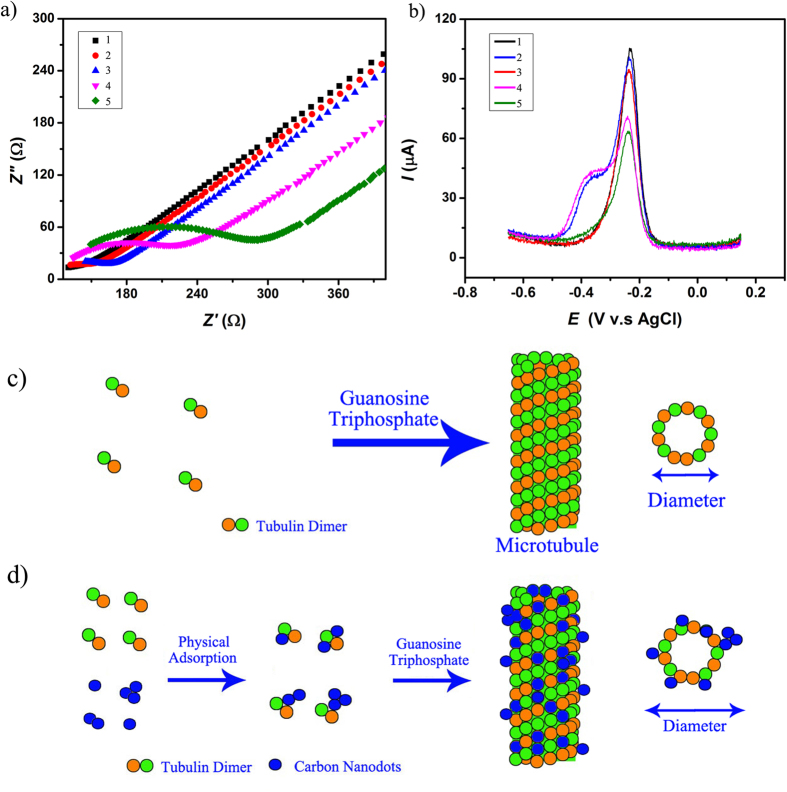
(**a**) Electrochemical impedance spectrum (EIS) graph of the electrode. 1. Bare gold electrode. 2. S-doped C-dots/Chitosan/Gold electrode. 3. Chitosan/Gold electrode. 4. S-doped C-dots-microtubule (MTs)/Chitosan/Gold electrode. 5. Microtubule (MTs)/Chitosan/Gold electrode. (**b**) Differential pulse voltammetry (DPV) graph of the methylene blue on different electrode. The color-coding is similar to what described in (**a**). (**c**) Scheme of microtubule polymerization from free tubulin under the chemical energy of guanosine triphosphate. (**d**) Scheme of the polymerization process of the S-doped C-dots hybrid microtubule; the schematic is not to scale.

Figure 5a shows the electrochemical impedance spectrum of a representative bare gold as well as of the functionalized electrode (where functionalization is refereed to the sample of choice), with the impedance recorded in potassium ferrocyanide. Previous investigations showed that potassium ferrocyanide is a suitable agent to help monitor surface functionalization of an electrode with Kafka *et al*. using the agent to distinguish non-complementary DNA in the electrochemical impedance spectrum^87^ and Moulton *et al*. to study electron transfer on the gold electrode before and after adsorption of serum albumin^88^.

Generally, the chitosan-based membranes had smaller impedances when they contained embedded S-doped C-dots or hybrids containing the S-doped C-dots thus confirming increased conductivities of such hybrids. Specifically, analyses showed that Nyquist plot^89^, representing the real and imaginary impedances plotted against each other for different perturbation frequencies and known to allow for the charge transfer at the electrode interface to be evaluated, was almost a straight line for the bare electrode (Fig. 5a; black curve, i.e., 1). This was attributed to the diffusion limited step of the electrochemical process at this electrode’s^90^ and thus indicated a very small value of its measured impedance. However, after chitosan or chitosan-containing embedded components immobilization, the Nyquist plot changed and the electrode impedance increased in a manner dependent with its functionalization. Specifically, the plot showed a larger semicircle corresponding to the larger electron transfer resistance for the S-doped C-dots-chitosan-gold electrode and S-doped C-dots-microtubule-chitosan-gold electrode. Complementarily, the impedance values of the S-doped C-dots-chitosan-gold electrode, microtubule-chitosan-gold electrode and S-doped C-dots-microtubule-chitosan-gold electrode, were about 180 Ω (blue; i.e., 3), 300 Ω (green; i.e., 5) and 240 Ω (pink; i.e., 4) respectively. The lower values of the obtained charge transfer resistances indicated a faster reaction rate at the interfaces with the C-dots nanodots or C-dots-based hybrids respectively, thus confirming the role of the C-dots in enhancing electron transfer at the membrane interfaces with the results being attributed to the fluent electron transfer between the potassium ferrocyanide and such membranes during the electrochemical reaction. Analyses also showed that, when compared with the chitosan-gold electrode, the membrane containing embedded S-doped C-dots in the chitosan membrane immobilized at the gold electrode had an additional peak with its impedance decreasing from 180 Ω to 150 Ω with the peak being a result of the additional species present during the S-doped synthesis and confirmed by the spectroscopy analyses.

The electrochemical behavior of the MB on the modified electrodes was also investigated as described previously. Analyses (Fig. 5b) confirmed that the peak current of the MB on the S-doped C-dots hybrid microtubule coated electrode (pink; i.e., 4) was larger than that on the control microtubule-coated electrode (green; i.e., 5) thus indicating that the electrochemical process came from the embedment of the C-dots. The largest peak current (about 105 μA) appeared to be on the bare gold electrode (black; i.e., 1), while the smallest peak current (about 65 μA) was recorded for the microtubule-chitosan-gold electrode (green; i.e., 5). Further, control experiments demonstrated that for hybrids and microtubules can be compared since there is no no-specific dissociation of the quantum dots from the former (Supplementary Information Figure S4).

Our analyses showed that all the curves contained peaks at about −0.22 V (vs Ag/AgCl) which confirmed the reduction of the MB cation (MB^+^)^91^, with an observed decrease from the gold, to S-doped C-dots-chitosan-gold electrode, chitosan-gold electrode, S-doped C-dots-microtubule-chitosan-gold electrode, to microtubule-chitosan-gold electrode respectively. In addition, there were two additional peaks at about −0.38 V on curve 3 associated with the S-doped C-dot-chitosan-gold electrode and 4 associated to the S-doped C-dot-MTs-chitosan-gold electrode respectively, with such peaks presumably originating from the additional chemicals contained in the S-doped C-dots as demonstrated by the EDX analyses. In particular, previous results have showed that the standard redox potential of SO_3_^2−^ is 0.571 V for instance, while according to the Nernst equation^92^ the redox potential of SO_3_^2−^ versus Ag/AgCl is 0.38 V thus being attributed to the reduction reaction of the SO_3_^2−^ and/or the reducing of SO_3_^2−^ to thiosulfuric (S_2_O_3_^2−^; Eq. 2).


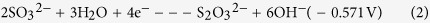


It is reasonable that the S-doped C-dots could act as a conducting bridge between the microtubule and the electrode to increase the conductivity of the hybrid structure, thus enhancing the electrochemical intensity effectively and further demonstrating the conductive features of the nanowire being created.

Nanowire structures are expected to offer user-controllable surfaces for novel functional hybrids with unique chemical and physical properties. For instance, studies by Xie *et al*. showed that silicon nanowire/carbon hetero junctions can be fabricated for the next generation of photovoltaic devices to show increased power conversion efficiency as resulted from the increased optical absorption at the C-dots interfaces^93^. Complementarily, Zhang *et al*. synthesized C-dots loaded TiO_2_ nanorods, with the C-dots serving as green sensitizers and showed that they are capable to enhance the hybrid material photo-responsiveness in visible light^94^.

Herein, the self-organization and self-assembly of tubulin onto thd user-synthesized S-doped C-dots was shown to lead to stable nanowire geometries of increased conductivities and proves to be a feasible alternative to the methods described in this paper’s introduction. Specifically, our hybrid microtubule nanowire of only about 14 nm in diameter and formed in mild conditions could allow for limited inhibition of the quantum confinement effect to thus lead to a functional structure. Further, such S-doped C-dots hybridized microtubule or bio-nanowire could prove to be an easy and “green” step in which new function can be implemented to a non-biological molecule to be recognized and transported to user-defined location by kinesin hangars. In particular, previous studies have showed that microtubule-based transport system, normally used in the cell for long distance transport of cargos, could be mimicked in synthetic environment to allow for transport of nanoparticles or nanotubes when kinesin molecular motors are used^43^. If such a transport can be implemented and observed for the S-doped C-dots hybrid microtubule, then one could envision not only the demonstrated assembly of functional nanowires with increased conductivities but further, their regular positioning under the energy control of adenosine triphosphate used to ensure kinesin biological function. Such approach has the potential to fully achieve the promising applications of such nanodots or resulting nanowires through both their control over the size as well as their orientation onto user-controlled surfaces and geometries^43^.

## Conclusions

In this study, we successfully synthesized a conductive and hybrid bio-nanowire by using the self-assembly properties of free tubulin (a biological molecule) and tubulin templated onto user-synthesized S-doped C-dots in solution. Our analyses showed that such nanowire is more conductive than control microtubule assembled only from free tubulin. Further, the nanowire had an average diameter of 14 nm that was superior (to our knowledge) to any of templated nanowires currently produced. Such synthesized bio-nanowire may increase the signal processing ability for the next generation of integrated circuits while ensuring regular positioning through biological self-rcognition capabilities.

## Additional Information

**How to cite this article**: Hu, X. *et al*. Protein self-assembly onto nanodots leads to formation of conductive bio-based hybrids. *Sci. Rep.* **6**, 38252; doi: 10.1038/srep38252 (2016).

**Publisher's note:** Springer Nature remains neutral with regard to jurisdictional claims in published maps and institutional affiliations.

## Supplementary Material

Supplementary Information

## Figures and Tables

**Table 1:** Percentage distribution of the elements present in the user-synthesized S-doped C-dots.

Element (%) Method	Carbon (C)	Oxygen (O)	Sodium (Na)	Sulfur (S)	Phosphor (P)
EDX	22.77	22.06	21.50	33.67	0
XPS	34.81	39.81	16.32	7.73	1.33
