# Supplementary material for: Protein self-assembly onto nanodots leads to formation of conductive bio-based hybrids
Source: Sci Rep. 2016 Dec 6;6:38252. doi: 10.1038/srep38252 (PMC5138619; doi:10.1038/srep38252)
Supplement: Supplementary Information [file srep38252-s1.pdf]

Supplementary Information

Protein self-assembly onto nanodots leads to formation of conductive bio-based hybrids

Xiao Hu[1], Chenbo Dong[1], Rigu Su[2], Quan Xu[2*], and Cerasela Zoica Dinu[1]*

[1]Department of Chemical Engineering, West Virginia University, WV, USA
[2]State Key Laboratory of Heavy Oil Processing, China University of Petroleum (Beijing), Beijing, China

***Corresponding authors:**
Cerasela Zoica Dinu, Ph.D.
Department of Chemical Engineering
West Virginia University
Benjamin M. Statler College of Engineering and Mineral Resources
PO Box 6102
Morgantown, WV, 26506, USA
E-mail: cerasela-zoica.dinu@mail.wvu.edu
Tel.: +1 304 293 9338
Fax: +1 304 293 4139

Quan Xu, Ph.D.
State Key Laboratory of Heavy Oil Processing
China University of Petroleum (Beijing)
Beijing, 102249,China
Email: xuquan@cup.edu.cn
Tel: +86-10-89731300
Fax: +86-10-89731300

1. Characterization of S-doped C-dots

The morphology of as-prepared S-doped C-dots was investigated using high-resolution transmission electron microscopy (HRTEM; Model JEM-2100). First, the samples (1 mL of purified S-doped C-dots solution was diluted into 10 mL of water), dropped onto copper wire meshes, and dried for 20 min at 55°C. An accelerated voltage of 200 KV was used for analyses. Sample's sizes are revealed in **Figure S1**.

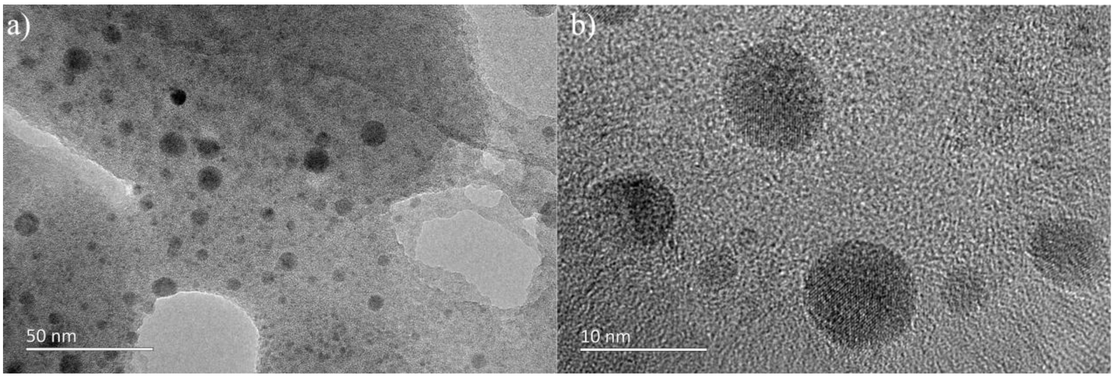

Figure S1: HRTEM images of S-doped C-dots. Scale bars: a) 50 nm, b) 10 nm.

2. High resolution AFM image of the S-doped C-dots

In order to provide details of the hybrids structure, high-resolution AFM images were obtained. The experimental details were exactly the same as described in the manuscript. Briefly, contact mode Atomic Force Microscopy (AFM, Asylum Research, USA) with a silicon nitride tip (TR-400PB, Asylum Research, USA) in solution was used. The trigger force was kept constant at 3 nN while the spring constant of the cantilever was measured before each experiment using established method.

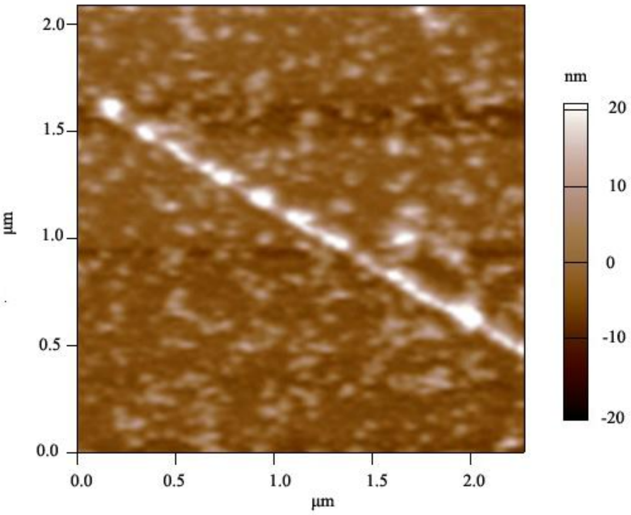

Figure S2: High resolution AFM image of the S-doped C-dots hybridized microtubule showing S-doped C-dots incorporated into the polymerized hybrid as a bead-like geometry.

3. Evaluate the effect of chitosan on tubulin self-assembly

The polymerization solution was obtained by vortexing 5 µL 100 mM magnesium

chloride ($MgCl_2$, Fisher Scientific, USA), with 6 µL dimethyl sulfoxide (DMSO, 99.7%, Fisher Scientific, USA), 5 µL 25 mM guanosine-5'-triphosphate (GTP, Sigma, USA) and 9 µL BRB80 buffer (formed from a mixture of 80 mM piperazine-N,N'-bis(2-ethanesulfonic acid buffer, 1 mM $MgCl_2$ and 1 mM ethylene glycol tetraacetic acid (EGTA), pH 6.8; all reagents were purchased from Fisher Scientific, USA).

Microtubule-chitosan were intended to be obtained upon mixing10 µL of 4 mg/mL rhodamine-biotin-tubulin with 10 µL 50% (wt) chitosan and initiating their polymerization. Specifically, 5 µL polymerization solution (see above) was injected into 20 µL of the rhodamine-biotin-tubulin-chitosan mixture and incubated at 37°C for 30 min. To stabilize any of the resulting structures, the solution was dispersed in 1 mL BRB80 buffer containing 10 µM taxol (Fisher Scientific, USA). The solution was kept at room temperature for experimental usage.

For the electrochemical impedance spectroscopy (EIS) analyses, 20 µL of the obtained "microtubule-chitosan" solution was dropped onto a cleaned electrode and incubated overnight under vacuum (same procedure as listed in the materials and methods). The EIS analyses have been performed in 50 mM potassium ferricyanide ($K_3Fe(CN)_6$; Fisher Scientific, USA) in BRB80 buffer containing 10 µM taxol. The supporting electrolyte used in our experiments is 10 mM NaCl.

Analyses reveal no major differences between the two curves (**Figure S3**).

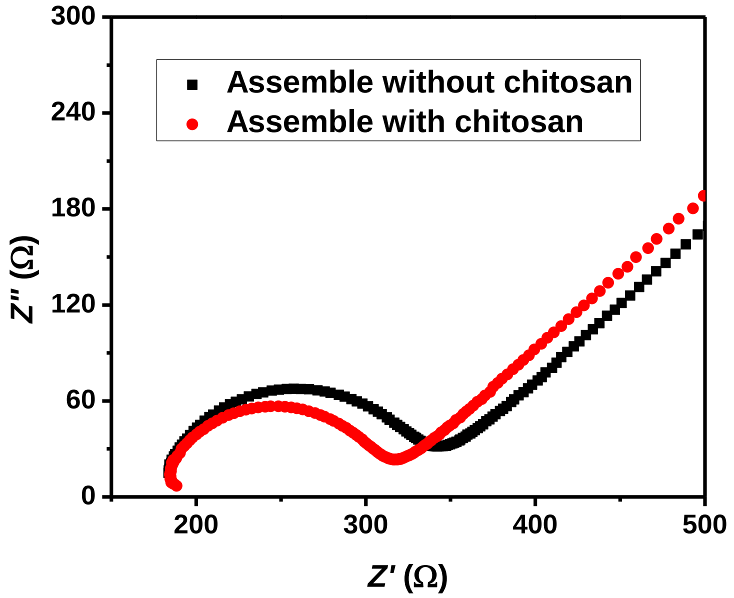

Figure S3: EIS graph of the modified electrode. Black curve: microtubule-chitosan/Au (the tubulin assembled in the presence of chitosan). Red curve: microtubule/chitosan/Au.

4. Evaluate the change in impedance on the chitosan membrane

For the bio-hybrid synthesis, also called hybrid microtubule, first

biotin-tubulin-S-doped C-dots-conjugates were formed using non-specific binding of biotin-tubulin onto S-doped C-dots scaffolds as described in the paper. Briefly, 1 µL 6 mg/mL synthesized S-doped C-dots were injected into a 600 µL eppendorf tube containing 5 µL of 4 mg/mL biotin-tubulin and the mixture was incubated for 2 h at 200 rpm in an ice bath. Subsequently, 5 µL of 4 mg/mL free biotin-tubulin was mixed with the biotin functionalized tubulin- S-doped C-dots conjugates and an additional 2.5 µL microtubule polymerization solution, and subjected to 37 ^{o}C for 30 min. When time elapsed, the hybrids were stabilized in BRB80 buffer containing 10 µM taxol.

Synthesized S-doped C-dots hybridized microtubules were spun down using high-speed centrifuge (30000 rpm for 10 min at room temperature). The supernatant was removed carefully and the pellet was re-suspended in 1 mL BRB80 buffer containing 10 µM taxol.

The synthesized microtubule and hybrids (before and after the centrifugation) were immobilized onto the electrode using the methods described in the manuscript. EIS analyses are presented in **Figure S4**; minor changes (not statistically relevant) have been observed most likely associated with changes in the microtubule/hybrid length known to occur because of the mechanical stress imposed by centrifugation.

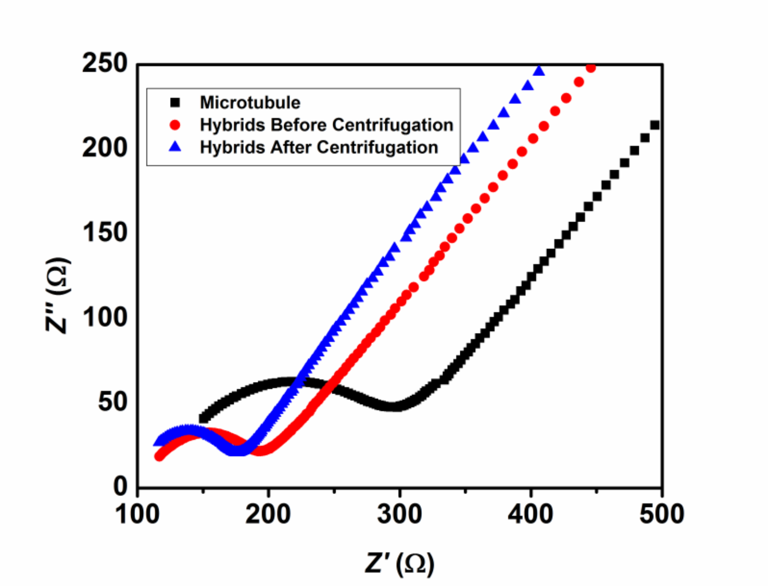

Figure S4: EIS graph of the modified electrode. Black curve: Microtubule/Chitosan/Au. Red curve: S-doped C-dots hybridized microtubule/Chitosan/Au. Blue curve, Centrifuged S-doped C-dots hybridized microtubule/Chitosan/Au.
